# Upconversion Spectral Rulers for Transcutaneous Displacement Measurements

**DOI:** 10.3390/s21103554

**Published:** 2021-05-20

**Authors:** Melissa M. Suckey, Donald W. Benza, John D. DesJardins, Jeffrey N. Anker

**Affiliations:** 1 Department of Chemistry, Clemson University, Clemson, SC 29634, USA; mmrogal@g.clemson.edu (M.M.S.); dbenza@g.clemson.edu (D.W.B.); 2 Department of Electrical and Computer Engineering, Clemson University, Clemson, SC 29634, USA; 3 Department of Bioengineering, Clemson University, Clemson, SC 29634, USA; jdesjar@clemson.edu; 4 Center for Optical Materials Science and Engineering (COMSET) and Environmental Toxicology Program, Clemson University, Clemson, SC 29634, USA

**Keywords:** spectral ruler, fluorescence, X-ray excited optical luminescence, autofluorescence, optical encoder, optical scattering, implanted medical device, sensing through tissue

## Abstract

We describe a method to measure micron to millimeter displacement through tissue using an upconversion spectral ruler. Measuring stiffness (displacement under load) in muscles, bones, ligaments, and tendons is important for studying and monitoring healing of injuries. Optical displacement measurements are useful because they are sensitive and noninvasive. Optical measurements through tissue must use spectral rather than imaging approaches because optical scattering in the tissue blurs the image with a point spread function typically around the depth of the tissue. Additionally, the optical measurement should have low background and minimal intensity dependence. Previously, we demonstrated a spectral encoder using either X-ray luminescence or fluorescence, but the X-ray luminescence required an expensive X-ray source and used ionizing radiation, while the fluorescence sensor suffered from interference from autofluorescence. Here, we used upconversion, which can be provided with a simple fiber-coupled spectrometer with essentially autofluorescence-free signals. The upconversion phosphors provide a low background signal, and the use of closely spaced spectral peaks minimizes spectral distortion from the tissue. The small displacement noise level (precision) through tissue was 2 µm when using a microscope-coupled spectrometer to collect light. We also showed proof of principle for measuring strain on a tendon mimic. The approach provides a simple method to study biomechanics using implantable sensors.

## 1. Introduction

Local biomechanical strain is a key factor for recovery (and reinjury) of musculoskeletal injuries. A degree of local strain encourages local tissue growth and repair, but too much strain can prevent healing, cause orthopedic hardware to fatigue, cause bones to fracture, and soft tissue tears to appear and propagate. Thus, detecting the small displacements associated with bone, soft tissue, or implanted hardware strain is important for understanding the injury and avoiding complications from improperly healing implants. For example, more than half the population over 60 years in age experiences a rotator cuff injury, and approximately 47 out of every 100,000 males between the ages of 10 and 19 years require anterior cruciate ligament (ACL) surgery each year [1,2,3]. However, direct measurement of the mechanical properties of tendon and ligaments remain elusive, and strain sensors are needed to elucidate these to monitor mechanics and develop treatments [1,4].

**Figure 1:**
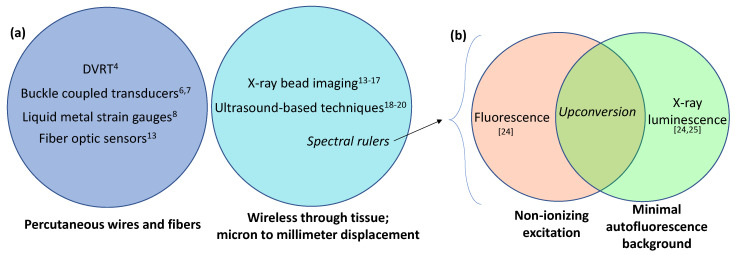
Venn diagram comparing several strain measurement techniques that could be applied to tendon strain measurement: (**a**) Wireless vs. percutaneous wires or fibers; (**b**) spectral rulers with three types of excitation/emission mechanisms.

Tendon strain is challenging to monitor in vivo in part because of location. Sensors can obstruct the function of the ligament/tendon; surrounding tissue and bone may press against the sensor leading to false readings, or the ligament/tendon is not in an easily accessible region. Refs. [4,5] Additionally, the strain distribution varies with position, and some studies have shown that currently available sensors and techniques are affected by loading rate and sensor rotation [1]. For ACL injuries, it is common to predict forces acting on the ligament with mathematical modeling [1,4], but this approach requires the scientist to make many anatomical and material property estimations or assumptions. Tendon strain has been monitored in vivo with the use of buckle transducers [6,7], liquid metal strain gauges [8], force and pressure transducers [9,10,11,12], fiber optic sensors [13], differential variable reluctance transducers (DVRT) [4], radiostereometric analysis (RSA) or radiopaque beads [14,15,16,17], and several ultrasound techniques, which track endogenous anatomical structures rather than implanted beads or markers. Refs. [18,19,20] each approach has its own advantages and limitations, relating to size, ease of application, maximum strain level, sensitivity, power, and readout mechanism (Figure 1).

Generally, optical strain and displacement measurements are advantageous because they can be performed noninvasively with simple equipment. However, mapping or imaging of a surface with micron-sized features becomes challenging when the tissue thickness is greater than ≈1 mm in depth because optical scattering in the tissue prevents light from maintaining collimation or focus and results in diffuse propagation with point spread functions typically larger than the tissue depth. Refs. [21,22,23] to overcome this limitation, we have developed an approach for measuring micron-scale displacements by collecting position-dependent luminescent signals. Using our methodology, small features do not need to be resolved; rather, position can be correlated with a change in color/wavelength of light emitted by a sensor film. The technique offers the advantage of noninvasive measurements, simplicity in data processing and interpretation of the signal readout, and the ability to measure position changes that would normally be unresolvable due to optical scattering. Our approach also eliminates the need for an ionizing radiation source.

Previously, we developed a fluorescence spectral-ruler-based displacement sensor that could be attached to a bone fracture to measure fracture gap movement or be placed on modified orthopedic screws to measure elongation under tension. Refs. [24,25] we also developed an X-ray excited luminescent spectral ruler that provided lower background interference but required an X-ray source, which added cost and radiation dose risks, especially for long-term or repeated measurements. Herein, we extend the principle to upconversion luminescence spectral rulers, which unlike X-ray excited luminescence use non-ionizing near infrared light as an excitation source, and unlike fluorescence do not have significant autofluorescent backgrounds and do not need multiple wavelengths to estimate and subtract off the background (Figure 1b). We apply the sensors to detection of strain observed in tendon mimics.

## 2. Materials and Methods

### 2.1. Chemicals and Materials

Gadolinium oxysulfide upconversion phosphors doped with ytterbium and erbium (4.0 micron PTIR660/F, Phosphor Technology LTD, Stevenage, UK) were purchased from Phosphor Technology Ltd. Sensors were coated with polydimethylsiloxane prepared from a Dow Corning Sylgard 184^®^ silicone elastomer kit (Elseworth Adhesives, Germantown, WI, USA). Bromocresol green dye (Alfa Aesar, Lancashire, UK), reagent alcohol (VWR BDH Chemicals, Randor, PA, USA), and dibasic sodium phosphate/monobasic potassium phosphate (BDH) buffer (pH 8) were purchased from VWR (Radnor, PA, USA). A Boise Aspen^®^ 300 92 bright copy paper (Boise Paper Holdings, L.L.C, Boise, ID, USA) was used for sensor fabrication, and 3 and 5 mil Fellowes laminating pouches (Enhance 3 mil photo card size laminating pouch and 5 mil business card size thermal laminating pouch, Fellowes, Itasca, IL, USA) were used to create the sensor housing. Multi-purpose transparency sheets were purchased from Staples (Product number 23240, Staples, Framingham, MA, USA). Bandpass filters were purchased from Semrock Inc.: the 661 nm filter had a 11 nm bandwidth, and the 680 nm filter had a 13 nm bandwidth. The 50/50 beam splitter was purchased from Chroma Technology.

Synthetic ligaments, Trevira (Kosa) artificial edge ligaments, were provided as a gift from Edge Medical Biologics (Manchester, UK).

### 2.2. Sensor Fabrication

Spectral encoders were designed in Inkscape, which is a freeware graphics program to contain 800 µm wide dye stripes with a 1.2 mm wide space between each. Bromocresol green was prepared at a concentration of 1.1 mg/mL in a 50/50 solution of pH 8 buffer (sodium and potassium phosphate) and reagent alcohol. The dye solution was added to a refillable ink cartridge and inserted into an Epson stylus R200 printer. For experiments performed using the collection optics of a fluorescent microscope, bromocresol green patterns were inkjet-printed onto copy paper using the CD tray insert. All patterns were printed a series of 9 times over the original pattern in order to increase the amount of dye transferred to the paper. Sensors were cut using a Graphtec CE6000 desktop cutter (Graphtec Irvine, CA, USA) to a sensor size of 15 mm × 60 mm. The sensor housing was constructed from 5 mil laminating pouches using a Fellowes laminator (Venus 125, Fellowes, Itasca, IL, USA) on the 4 mil heat setting. The analyzer mask pattern was designed in Inkscape to contain 1 mm wide opaque stripes with a 1 mm wide space between each. The analyzer was printed on the smooth (non-polymer coated) side of a Staples transparency sheet using an HP Color LaserJet 1518ni printer (HP Inc., Palo Alto, CA, USA) in black ink. The opaque stripes were printed 200 µm wider than the dye stripes on the encoder to accommodate for the ink spreading observed on paper substrates with the inkjet printer. The sensor housing was attached using double-sided tape to an upconversion microparticle film prepared on a foil sheet. The film dimensions were 25 mm × 5 mm and contained 260 µL of a PTIR660/F solution (0.17 g/mL PTIR660/F in an aqueous solution containing 5 mg/mL carboxymethyl cellulose sodium salt). The particle film was prepared by drop coating and was left to dry at room temperature overnight.

For samples with luminescence measured using photomultiplier tubes, the sample preparation was modified. An interdigitated sensor pattern was designed in Inkscape containing interlocking 950 µm wide stripes. In place of copy paper, a block of bromocresol green was printed onto a transparency sheet using the CD tray insert. The block was printed over an additional 9 times. The interdigitated design was cut from the dye-printed transparency as well as from a blank transparency sheet. A blank and a dye containing a sensor half were fit together and laminated inside a 3 mil laminating pouch. Then, the sensor’s dimensions were cut to 12.4 mm × 21.0 mm, double-sided tape was attached to one side, and the encoder was fit inside an empty encoder housing prepared from a 5 mil laminating pouch. The analyzer mask was printed with the HP Color LaserJet 1518ni printer to contain alternating black dye and transparent spaces of equal width (950 µm). The sensor was overlaid upon a 6 mm × 18 mm upconversion particle film containing 250 µL of 0.72 mg/mL PTIR 660/F in an aqueous solution containing 5 mg/mL carboxymethyl cellulose sodium salt.

While the purpose of the prototype was to show proof of principle rather than in vivo work, the materials are generally suitable for in vivo work. Specifically, the Gd_2_O_2_S upconversion phosphors are relatively well tolerated even when injected intravenously as nanoparticles up to at least 75 mg/kg in mouse models [26] and 400 mg/kg in rat models [27], and encapsulating microparticles into films would be expected to have lower toxicological profile (indeed, encapsulation is a common way to provide biocompatibility of even toxic implanted materials such as in liquid metal strain gauges and batteries); carboxymethyl cellulose is generally considered biocompatiable and has been used in mammary implants without ill effect [28]; the transparencies are made from cellulose acetate, which has been applied in a number of biomedical applications for drug release, tissue regeneration, and biosensors [29]; the paper is made from cellulose fibers and paper has been proposed for tissue scaffolds [30,31]; the laminating pouch is made from an inner hot-melt adhesive layer of ethylene-vinyl acetate (EVA) (generally considered biocompatible for some implant applications [32]), and an outer layer of polyethylene terephthalate (PET), which has been used ACL repair in sheep studies [33], and the surface can be modified with hydroxyapatite for osteointegration applications [34].

### 2.3. Luminescent Measurements

**Figure 2:**
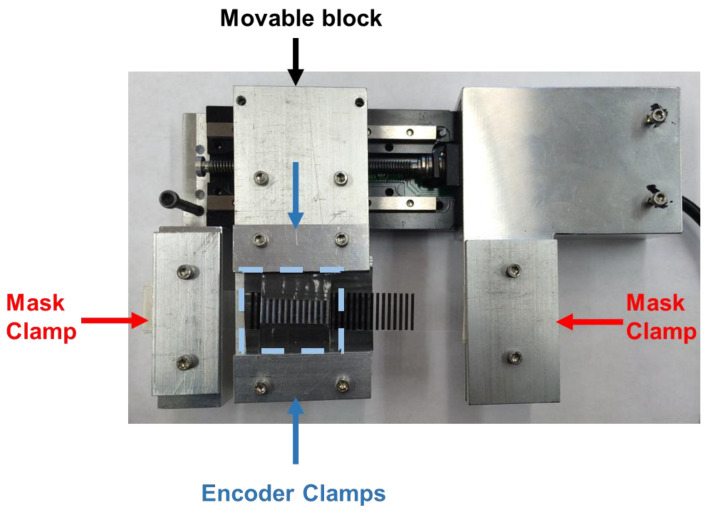
Photograph of an upconversion spectral ruler held by the motorized stage fixture. The analyzer mask is held in a fixed position, clamped on either end. The encoder and particle film are attached to a metal extension screwed into the movable portion of the motorized stage. The encoder is highlighted in the light blue dashed box.

For all luminescent measurements, the spectral encoder housing was clamped to a mechanical stage with a machined fixture prepared to fit an MTS 50-Z8 stage (Thorlabs, Newton, NJ, USA) (Figure 2). The analyzer mask was fixed at both ends to maintain the substrate in a stationary position. The backlash correction of the software program, ThorLabs APT user (Version 3.2.5697.24155), was 0.05 mm. The functionality and reproducible motion of the stage was previously characterized. Briefly, the backlash for 25, 35, 50, and 100 µm displacements was determined to be 10.5 ± 0.7 µm, 10.7 ± 0.5 µm, 17.8 ± 0.9 µm, and 18.7 ± 0.8 µm respectively. Displacements were adjusted accordingly for changes in stage direction.

For luminescent measurements for sensors prepared on copy paper, the mechanical stage was inverted and placed over a 10× microscope objective of an inverted fluorescence microscope (DMI 5000, Leica Microsystems, Germany). A 3 mil laminating pouch was placed between the sensor and the microscope objective. The sensor was positioned above the collection optics and the objective height was set to 8.60 mm (the minimum distance allowable between the objective lens and sample plane). The spectral ruler was excited from below with a 500 mW, 980 nm laser (Changchun New Industries Optoelectronics Technology, Changchun, China) after the laser emission was filtered through a 980/15 nm bandpass filter (Chroma Technology Corp, Bellows Falls, VT, USA). Sensor luminescence was passed through a 960 nm short pass emission filter (Chroma Technology Corp, Bellows Falls, VT, USA) prior to being sent to a CCD camera (iDUS-20BV, Andor, South Windsor, CT, USA) in a DeltaNu spectrometer (DNS 300, DeltaNu, Laramie, WY, USA). The efficiency of the light collection was improved with the addition of a cylindrical lens between the microscope and spectrometer. Spectra were collected with either a 150 line/mm grating centered at 600 nm or a 1200 line/mm grating centered at a value of 1290. For the grating with finer spacing, the pixel number readout of the spectrometer was calibrated using room light to convert the values into nanometers. Using Andor Solis software (Version 4.242.30004.0), the collection exposure time was set to 0.30 s for the 150 lines/mm grating and 0.050 s for the 1200 lines/mm grating. Spectra were recorded at a readout rate of 100 kHz with 16-bit resolution corresponding to a signal output to photoelectron conversion of 15 photoelectrons per count.

Luminescence measurements were acquired using a portable collection system for the interdigitated sensors. The spectral ruler was attached to the motorized stage and placed on the reflective surface of a lab jack to adjust sensor height. An optical fiber with attached lens was positioned to illuminate the spectral ruler from above with a portable 500 mW, 980 nm laser filtered through a 980/15 nm bandpass filter (Chroma Technology Corp, Bellows Falls, VT, USA). A collection lens was centered above the ruler and illuminated area. At the exit of the lens, a 3 ft long, 5 mm diameter liquid lightguide was attached to transfer the signal to a collimator at the entrance of a filter cube. The light was passed through an 842 nm short pass filter to ensure no bleed through from the laser (if not properly excluded by the 980 nm filter) would be collected and influence calculated spectral ratios. Then, the light was redirected by a 50/50 beam splitter and passed through either a 661/11 nm bandpass or 680/13 nm bandpass emission filter. At the exit of each lens was a photomultiplier tube (Sens-Tech P30PC-01 Berkshire, UK) with a 25 mm collection area. Photon counting was performed using a USB-interfaced data acquisition device (model NI 9402 National Instruments, Austin, TX, USA). Spectral acquisition settings were controlled with a custom LabView program. The signal was acquired with a sampling frequency of 1 MHz and acquisition time of 100 milliseconds. To ensure that the luminescent signal did not saturate the number of counts that could be processed by the data acquisition device, a sheet of white paper was placed between the sample and the excitation source and collection optics to reduce the signal. A total of 100 measurements were acquired at each stage position, and the intensity readouts were averaged for each location. The same motorized stage used for the microscope measurements was used for the portable set up and controlled with ThorLabs APT software. Motorized stage step sizes were programmed to be either 50, 35, or 25 µm. Backlash corrected measurements correspond to actual distances of 32.2 ± 0.9 µm, 24.3 ± 0.5 µm, and 14.5 ± 0.7 µm respectively upon change in direction of stage motion. For luminescent measurements acquired through tissue, a 1.0 s exposure was used, and the sample was placed below a 6 mm slice of chicken breast tissue wrapped in cling wrap. Spectral ratios (I_661/_I_680_) were calculated after subtraction of a background (dark counts of the collection system).

## 3. Results and Discussion

### 3.1. Sensor Operation

**Figure 3:**
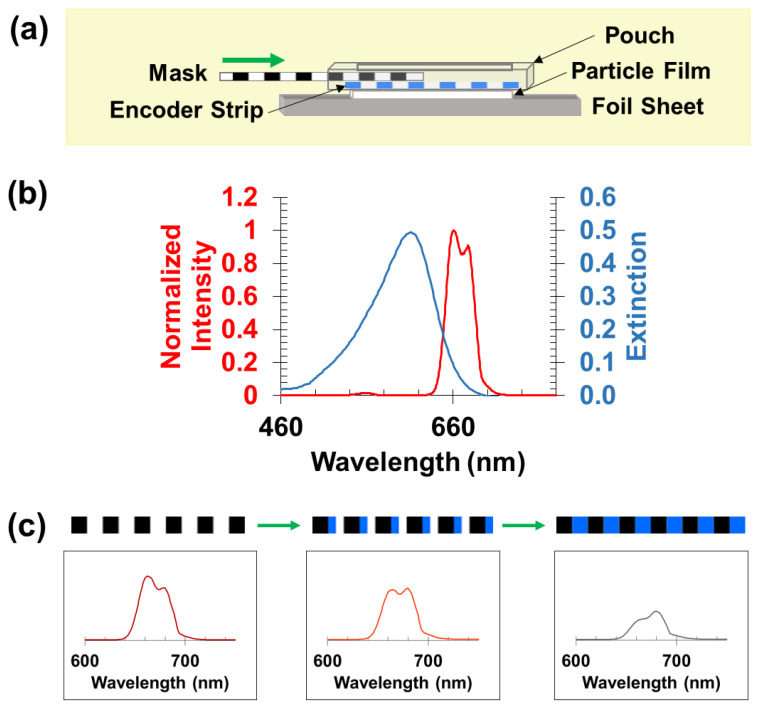
(**a**) Schematic of the spectral ruler sensor design showing assembly of the sensor inside a semi-rigid laminating pouch. The sensor assembly overlays an upconversion microparticle film. (**b**) Absorbance spectrum of bromocresol green (pH 8) in solution overlaid upon the emission spectrum of a Gd_2_O_2_S:Yb,Er film excited with 980 nm light. (**c**) Three distinct positions of the encoder with respect to the analyzer mask are shown with their corresponding emission spectra. As the analyzer windows move and reveal a larger fraction of dye-covered phosphors, the less low wavelength emission is detected compared to higher wavelength emission.

The sensors evaluate position by monitoring a luminescent signal generated from upconversion microparticles below an encoder strip. The encoder strip contains alternating lines printed with a dye that absorbs red light. The encoder is overlaid with a mask, which is referred to as the analyzer. The analyzer is a transparent film patterned with a black dye with line spacing equal to the lines on the encoder. The black dye does not transmit light produced by the microparticle film. Both the encoder and the analyzer’s motion are constrained to a single axis by confining the encoder strip inside a semi-rigid laminate pouch containing guiding rails that restrict the motion of the overlaid analyzer. Figure 3a depicts a schematic of the sensor design, showing the microparticle film below the housing of the encoder strip. A luminescent signal is generated by the sample when irradiated with a 980 nm laser, exciting the microparticle film. The luminescent signal passes through the encoder layer of the sensor before exiting through the analyzer mask and traveling to the detector (spectrometer). The signal output is modulated based on analyzer position; when the blackened regions of the analyzer overlay the dye on the encoder, the spectrum collected represents that of the upconversion microparticles. Alternatively, when the dye region of the encoder is fully revealed through the analyzer, a portion of the luminescent signal from the upconversion particles is absorbed by the dye. Luminescence observed at three distinct alignments of the analyzer over the encoder strip is shown in Figure 3c along with the absorbance profile of the dye overlaid upon the emission spectrum of the microparticle film (Figure 3b). When the analyzer is in an intermediate position between the two extremes described, the spectrum observed is a linear combination of the start and end positions.

The upconversion spectral rulers used here had a spectral encoder consisting of 980 nm excited Gd_2_O_2_S:Yb,Er upconversion microphosphors in polydimethylsiloxane (PDMS) and covered with alternating lines of bromocresol green (BG) dye. These lines of dye altered the emission spectrum by absorbing the 680 nm light more strongly than 670 nm or 660 nm. Thus, the 660 nm/680 nm intensity ratio depends upon the analyzer position (see Figure 1b). For calibration purposes, we attached our encoder housing to a motorized stage with the analyzer mask attached separately on either end to hold its position fixed relative to the microscope. Figure 2 shows a photo of the setup. The motorized stage position was electronically controlled using ThorLabs APT software.

Previously, we showed the concept of absorbance of luminescence from an Er^3+^ doped upconversion film by bromocresol green with the development of a pH sensor capable of effectively measuring pH between pH 5 and 10. At low pH values, there is minimal overlap of the dye absorbance with the particle emission peak above ≈650 nm. Spectral ratios were calculated for emission intensities at 661 nm and 671 nm after excitation with 980 nm light. A power dependence study for the emission wavelengths revealed that ratios calculated from these closely spaced peaks would not be affected by laser intensity. With this study, luminescent signals were collected through ≈6–7 mm of porcine tissue and pH monitored during the growth of *S. epidermidis* bacteria [35]. In the displacement (or strain) sensor used here, the dye was deposited at pH 8, dried, and sealed so it did not respond to pH and was only used to make the spectral encoder.

We also demonstrated a similar principle with spectral rulers patterned with bromocresol purple overlaying a Gd_2_O_2_S:Eu X-ray scintillator film and with an extension of the pH sensors described above [36]. With the XEOL version of the spectral ruler, we demonstrated that 14.5 µm changes in position could be detected through a tissue depth of 6 mm. Signals were collected through chicken breast tissue and were shot noise limited. However, these required an X-ray source, which was relatively expensive compared to a 980 nm laser; also, the risk of damage from ionizing radiation for the X-ray source precludes long-term or repeated analysis [24,25]. Similarly, we showed a fluorescence-based spectral ruler that did not use ionizing radiation but had significant interference from tissue autofluorescence (similar in magnitude to the signal from the spectral ruler for the conditions tested) [24]. To account for autofluorescence, we fit the raw spectra to a linear combination of spectra from the two spectral ruler quantum dots and autofluorescence from another slice of chicken breast. However, this approach required measurements at multiple wavelengths (in this case, a spectrometer) and would not have allowed a simpler two-wavelength measurement. In addition, it assumed that the sample had a fixed autofluorescence that did change with depth and time, which would be hard to ensure in future in vivo applications, especially for sensors in deeper tissue and with skin, where autofluorescence would be more intense, spectral distortion would be more significant, and the spectral ruler signal would be more attenuated. Fortunately, there are several ways to reduce or avoid autofluorescence (e.g., using lifetime gating on long lifetime phosphorescence materials, X-ray excited luminescence, or upconversion luminescence).

Upconversion particles offer several advantages over conventional fluorescence approaches for generating a luminescent signal to be collected through tissue. Upconversion nanoparticles are typically excited with lasers with output in the infrared, such as 980 nm light. Light of this wavelength (980 nm) is well suited for tissue studies because the relatively low optical absorption and scattering in the tissue allow it to penetrate deeply into tissue [37,38,39,40]. Additionally, there is minimal background from two photon autofluorescence because tissue autofluorescence requires essentially the simultaneous absorption of two photons (very high power, usually from pulsed laser sources); by contrast, our upconversion phosphors employ the sequential absorption of excitation light, which provides a much higher cross-section at the excitation powers used [41].

**Figure 4:**
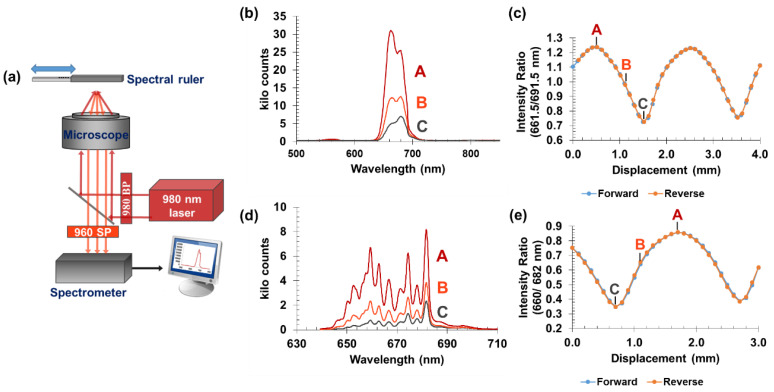
(**a**) Experimental set-up schematic. The spectral ruler is attached to a motorized stage and positioned above the microscope objective. The sample is excited from below with a 980 nm laser, and the luminescent signal generated is captured by a spectrograph with a 0.30 s exposure time. (**b**) Luminescent spectra acquired using a low-resolution 150 line/mm grating at three distinct encoder positions, which are labeled A (with no BG dye observed through the mask), B (intermediate position), and C (maximum BG dye observed through mask). (**c**) The 661.5/691.5 nm spectral ratio versus encoder position, including the labeled positions corresponding to A, B, and C in (**b**). (**d**) High-resolution spectra with 1200 line/mm grating at three distinct encoder positions, which are labeled A (with no BG dye observed through the mask), B (intermediate position), and C (maximum BG dye observed through mask). (**e**) The 661/682 nm spectral ratio versus encoder position, including the labeled positions corresponding to A, B, and C in (**d**). Backlash correction for (**c**,**e**) are shown in Figure S1.

Initial experiments involving our upconversion sensor were conducted using an inverted fluorescence microscope stage with a 10× microscope objective to collect the luminescent signal. With this set-up (Figure 4), there is significant light loss resulting in a low collection efficiency due to the small collection angle of the microscope objective and the dimensions of the spectrometer slit width.

Experimental results from these measurements demonstrate proof of concept. Figure 4b shows the luminescent signal acquired with a 0.30 s exposure and a spectrometer grating with a spacing of 150 lines per mm. As expected, an increase in the amount of bromocresol green dye visible through the transparent windows of the analyzer mask resulted in a greater absorption of the luminescent signal at 661 nm in comparison with luminescence at ≈691 nm (left to right side of the emission peak). We calculated spectral ratios for each unique encoder position; each data point represents a 100 µm displacement of the encoder. Finer spectral details were observed by repeating the calibration with a grating containing 1200 grooves per mm. The spectra in Figure 4d show the luminescent signal for the finer grating spacing and the calibration curve can be seen in Figure 4e. It was observed that there was a small offset between the forward and reverse directions corresponding to backlash; when a 12 µm backlash correction was applied, the curves overlap within 2 µm (Supplementary Material Figure S1).

### 3.2. Fiber-Coupled PMT-Based System

**Figure 5:**
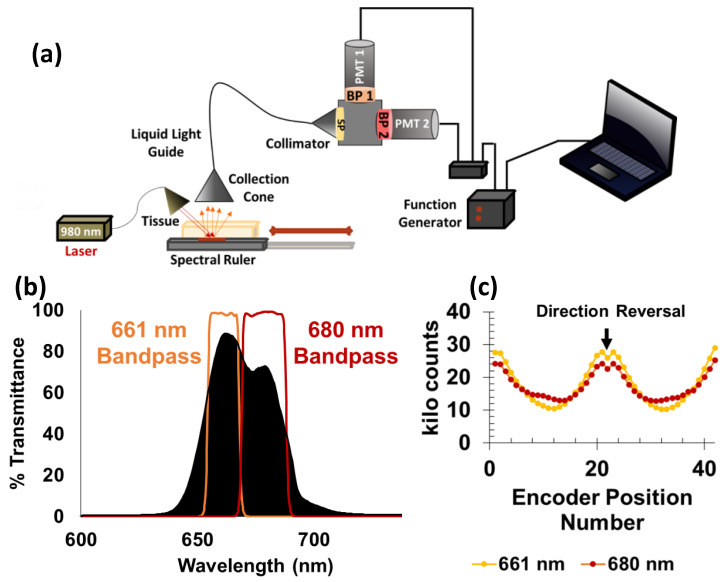
(**a**) Schematic of the fiber guided experimental setup. The sensor is excited with fiber-coupled 980 nm light, and the emission is collected with an optical fiber that passes through an 842 nm short-pass filter before reaching a 50/50 beam splitter housed within a filter cube. The beam splitter directs the light through either one optical arm with a 661 nm bandpass and PMT photodetector or a second arm with a 680 nm bandpass and second PMT. (**b**) Sensor upconversion spectrum overlaid with bandpass filter transmission spectra. (**c**) Average intensity vs. encoder position (a 100 µm encoder displacement was performed between each position). Each data point calculated from the average of a hundred 1.0 s measurements at a fixed position. The motorized stage direction was reversed after 2.0 mm of travel. Zoom in of 5c with error bars is shown in Supporting Info Figure S2a.

To improve our instrument’s collection efficiency and make it portable for eventual use in a clinical setting, we used photomultiplier tubes in place of the microscope optics and CCD camera (see Figure 5). The modified set-up also offers the advantages of being cheaper and simpler and provides the possibility for increased data collection speed. No modifications were made to spectral ruler attachment to the mechanical stage (pictured in Figure 2) and the operation of the stage control software. The ruler was illuminated from above with a 980 nm laser, and the microparticle emission was captured with a collection lens (focal length 40 mm ± 5%, a numerical aperture of 0.55, and a lens diameter of 50 mm) before traveling through a 3 ft long liquid light guide (5 mm diameter) to a collimator attached to the entrance of a filter cube. The emission was first passed through an 842 nm short-pass filter to ensure no excitation light would reach the detectors. After passing through the filter, the emission was directed to a 50/50 beam splitter. Beyond the beam splitter were two separate bandpass filters (emission maxima at 661 or 680 nm) and a photomultiplier tube for each. Figure 5b shows the filter transmission spectra.

Sensor preparation was also modified to increase the low signal output observed for measurements through tissue using the initial set-up. For the microscope experiments described earlier, the encoder strip was prepared on white copy paper with a thickness of ≈100 µm. White paper is a highly scattering medium and therefore was replaced with a transparent film to decrease signal scattering and increase the amount of light detected. With paper as the encoder substrate coupled with the small collection angle of the microscope objective, it was challenging to achieve a sufficient signal through tissue. This modification was made for all sensors prepared for measurements using the PMT collection system reported here.

**Figure 6:**
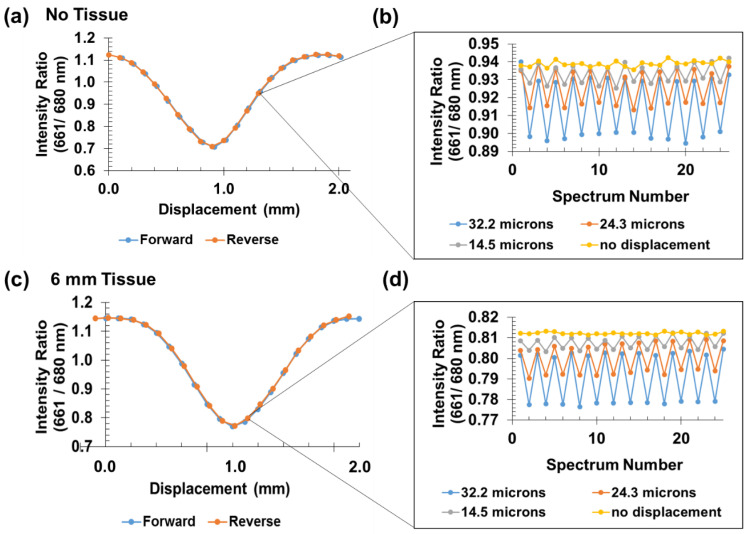
Sensor response over 2 mm displacement and small cyclic displacements measured with and without tissue. (**a**,**c**) show spectral ratio (I_661_/I_680_) vs. sensor displacement for a travel distance of 2.0 mm. Each point represents a 100 µm displacement. (**b**,**d**) show reproducible spectral ratios for a single position (single forward displacement followed by a return to the initial position). All displacement measurements in (**a**,**c**) were corrected for backlash of the motorized stage. Measurements were acquired with a 1.0 s exposure.

A calibration curve for sensor displacement in the absence of tissue was generated using the new collection system. To reduce signal intensity and avoid saturating the PMTs (given the much better collection efficiency compared to the microscope-coupled spectrometer), white paper was placed over the top of the spectral encoder to attenuate and scatter both the 980 nm excitation and emitted upconversion luminescence. Each data point on the calibration curve represents the average of 100 measurements acquired at a single position. Average intensities vs. encoder position for each bandpass filter are provided in Figure 6c. Spectral ratios (I_661_/I_680_) were calculated from the average intensity values. Figure S2 in the Supporting Information shows the encoder position data and corresponding ratio. In this case, while there are significant differences between forward and reverse intensities (likely due to fluctuations in laser intensity and tissue transmittance), the ratio curves overlap well, indicating that the spectral ratio accounts for most of this common mode noise.

### 3.3. Small Signal Analysis

We next tested the sensor’s ability to measure reproducible displacements on the tens of microns scale. Single forward and reverse motions of the mechanical stage revealed that 14.5 µm displacements could be measured in the absence of tissue. Each data point was determined from the average of 100 measurements at that position. This averaging reduced the theoretical random uncorrelated noise by a factor of 10 for this dataset.

The average percentage error for all step sizes excluding 0 µm (no motion) was calculated to be 0.18%. We converted the noise level on the signal to an error in displacement using the average difference in spectral ratio for a 32.2 µm displacement divided by the stage step size. A change in ratio of 9.93 × 10^−4^ at this stage position corresponds to a displacement change of 1 µm. For measurements acquired in the absence of motion (0 µm step size), the percentage error agreed with the average error for the three steps sizes reported, 0.18%, and suggests the data is shot noise limited. Given the linear response in this region, the uncertainty in intensity ratio corresponds to a 1.7 µm uncertainty in displacement. Although measurements for step sizes less than 14.5 µm were not attempted due to the backlash limitations of the motorized stage, we expect that we can detect displacements of smaller magnitude based on these error analyses.

We repeated the calibration curve measurements through tissue; 6 mm of chicken breast tissue was placed above the sample. To ensure that a significantly bright signal was acquired, we increased the acquisition time from 100 milliseconds (in the absence of tissue) to 1.0 s. The calibration curve is in good agreement with our previous measurements in the absence of tissue. A single displacement study was performed through tissue using the same programmed motorized stage step sizes as shown in Figure 6b. Although the signal intensity was decreased through tissue, we measured 14.5 µm displacements with low position error. The calculated noise associated with error in position was determined to be 0.73 µm when there was no stage displacement. Figure S3 shows the raw data with 2500 one-second measurements with no displacement, the mean and standard deviation of 25 sets of 100 of the measurements averaged, and the ratio and standard deviation of these datasets. There was around 0.15% fluctuation in both 661 and 680 nm intensity during the acquisition, but these were correlated (likely due to common mode variation in laser intensity), and the ratio was shot noise limited (Figure S3). The noise for cyclic loading measurements had an average of 2.0 µm variation for the 14.5, 24.3, and 32.2 µm displacements. We attribute the small increase in error to buckling of the analyzer mask and imprecision in stage control/motion of the encoder.

In addition to showing proof of principle for through tissue displacement measurements, we have also shown that we can monitor displacements at a less sensitive region of the calibration curve. The small displacement study was conducted at the steepest slope of the linear portion of the curve, while the through-tissue measurements were collected close to a sensor position extreme (almost 100% of the region below the transparent window containing bromocresol green dye).

In this study, we have shown that we can image through 6 mm of chicken breast. We do not know the maximum but would expect around 2 cm should be feasible because we are able to acquire focused X-ray luminescence images through around 2 cm of tissue and bone in an ex vivo rabbit model [42], and we typically observe much higher signals from upconversion luminescence than for X-ray luminescence [43]. However, deeper tissue imaging may require better optical collection efficiency, larger or brighter sensors, longer acquisition times, and/or higher noise levels because the excitation and emission light are exponentially attenuated by tissue.

### 3.4. Tendon Measurements

**Figure 7:**
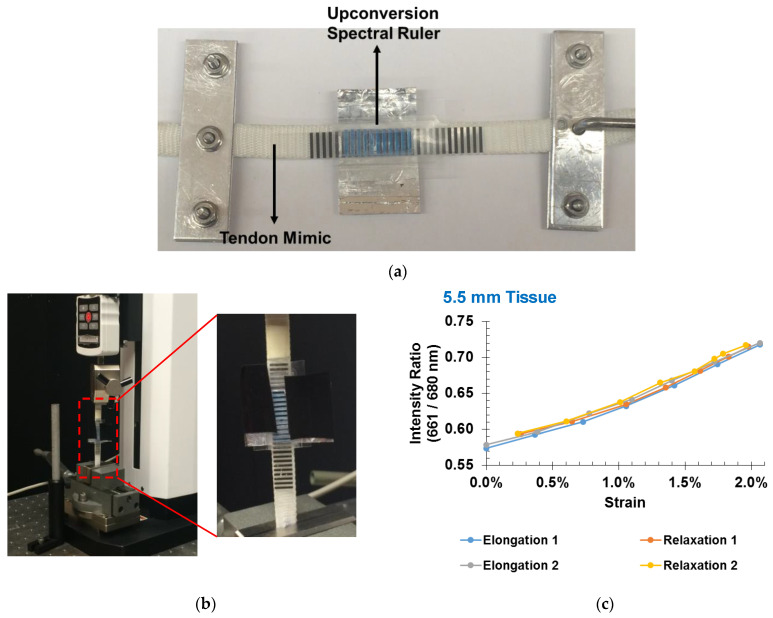
(**a**) Photograph of an upconversion spectral ruler attached to a 10 mm wide cruciate ligament mimic. (**b**) Photo of setup on Mark-10 test stand. (**c**) Spectral ratio vs. strain for load cycles, measured through 5.5 mm of chicken breast tissue. Each point represents 10 s of data (the average of 10 measurements with 0.1 s exposure each).

To show a potential application, we attached the spectral ruler to a 10 mm wide cruciate ligament mimic. A photograph of the ligament with an attached sensor is provided below in Figure 7. The initial gauge length (distance between points of attachment) was measured. We applied known forces to elongate the ligament using a force testing system. The luminescent signal generated by the ruler was collected at each force reading. The signal was viewed through 5.5 mm of tissue and increased with strain between 0 and 2%, with reproducibility of around 0.1% strain.

## 4. Conclusions and Future Work

We demonstrated proof-of-concept for measuring reproducible micron-scale displacements through tissue using a novel upconversion spectral ruler. The rulers provide a low noise, low background signal through tissue without the use of ionizing radiation. We have measured displacements ranging from 100 µm down to 14.5 µm with signals limited primarily by shot noise and displacement step size limited by the backlash of the motorized stage. We expect that we can measure displacements as small as ≈2.0 µm through tissue. By detecting a change in wavelength/color, the method does not require small features to be resolved through a biological medium. We have also demonstrated the ability to collect measurements with a simple and portable collection system. Future work will include miniaturization of the sensors to be applied in the measurement of strain on tendons/ligaments in animal models. We will approach the miniaturization using photolithography or 3D printing with custom filaments prepared by our lab containing luminescent materials.

## 5. Patents

The underlying invention is patented in Anker J.N., Rogalski M., Anderson D. and Heath, J.: “Luminescent Tension-Indicating Orthopedic Strain Gauges for Non-Invasive Measurements Through Tissue.” US Patent US20140046191 A1.

## Data Availability

The data supporting the findings of this article available within the article and Supporting Information.

## Supplementary Materials

The following are available online at https://www.mdpi.com/article/10.3390/s21103554/s1, Figure S1: Spectral intensity ratio acquired using a 150 line/mm diffraction grating vs. displacement (a) without backlash correction and (b) with a 12 µm backlash correction. Spectral intensity ratio acquired using a 1200 lines/mm diffraction grating vs. displacement (c) without backlash correction and (d) with a 12 µm backlash correction. Figure S2: Displacement measurements through 6 mm chicken breast tissue. (a) Raw intensities at 661 nm and 680 nm, same data as Figure 5c. (b) Replotted as intensity vs. position. (c) Calculated Intensity ratio (also shown in Figure 6c). Figure S3: Spectral ruler measurements through 6 mm of chicken tissue, used to generate Figure 5d. (a) Intensity at 661 nm and 680 nm (1 s exposure for each measurement). (b) Intensity at 661 nm and 680 nm for averaging 100 measurements. (c) Ratio of 661 nm/680 nm intensities (1 s exposure for each measurement). (d) Ratio of 661 nm/680 nm intensities for averaging 100 measurements.
