# Supplementary material for: Upconversion Spectral Rulers for Transcutaneous Displacement Measurements
Source: Sensors (Basel). 2021 May 20;21(10):3554. doi: 10.3390/s21103554 (PMC8160897; doi:10.3390/s21103554)
Supplement: Supplementary file 1 [file sensors-21-03554-s001.zip › sensors-1187991-supplementary.pdf]

Supplementary Materials

Upconversion Spectral Rulers for Transcutaneous Displacement Measurements

Melissa M. Suckey [1], Donald W. Benza [1,2,†], John D. DesJardins [3] and Jeffrey N. Anker [1,3,4,*]

[1] Department of Chemistry, Clemson University, Clemson, SC 29634, USA; mmrogal@g.clemson.edu (M.M.S.), dbenza@g.clemson.edu (D.W.B.)
[2] Department of Electrical and Computer Engineering, Clemson University, Clemson, SC 29634, USA;
[3] Department of Bioengineering, Clemson University, Clemson, SC 29634, USA; jdesjar@clemson.edu
[4] Center for Optical Materials Science and Engineering (COMSET) and Environmental Toxicology Program, Clemson University, Clemson, SC 29634, USA
* Correspondence: janker@clemson.edu
† Present address: Savannah River National Labs, Jackson, SC 29831, USA

Contents:

Figure S1. Spectral intensity ratio versus displacement with and without a 12 μm backlash correction. (Page 2)

Figure S2. Displacement measurements through 6 mm chicken breast tissue. (Page 3)

Figure S3. Spectral ruler measurements through 6 mm of chicken tissue, used to generate Figure 5d. (Page 4)

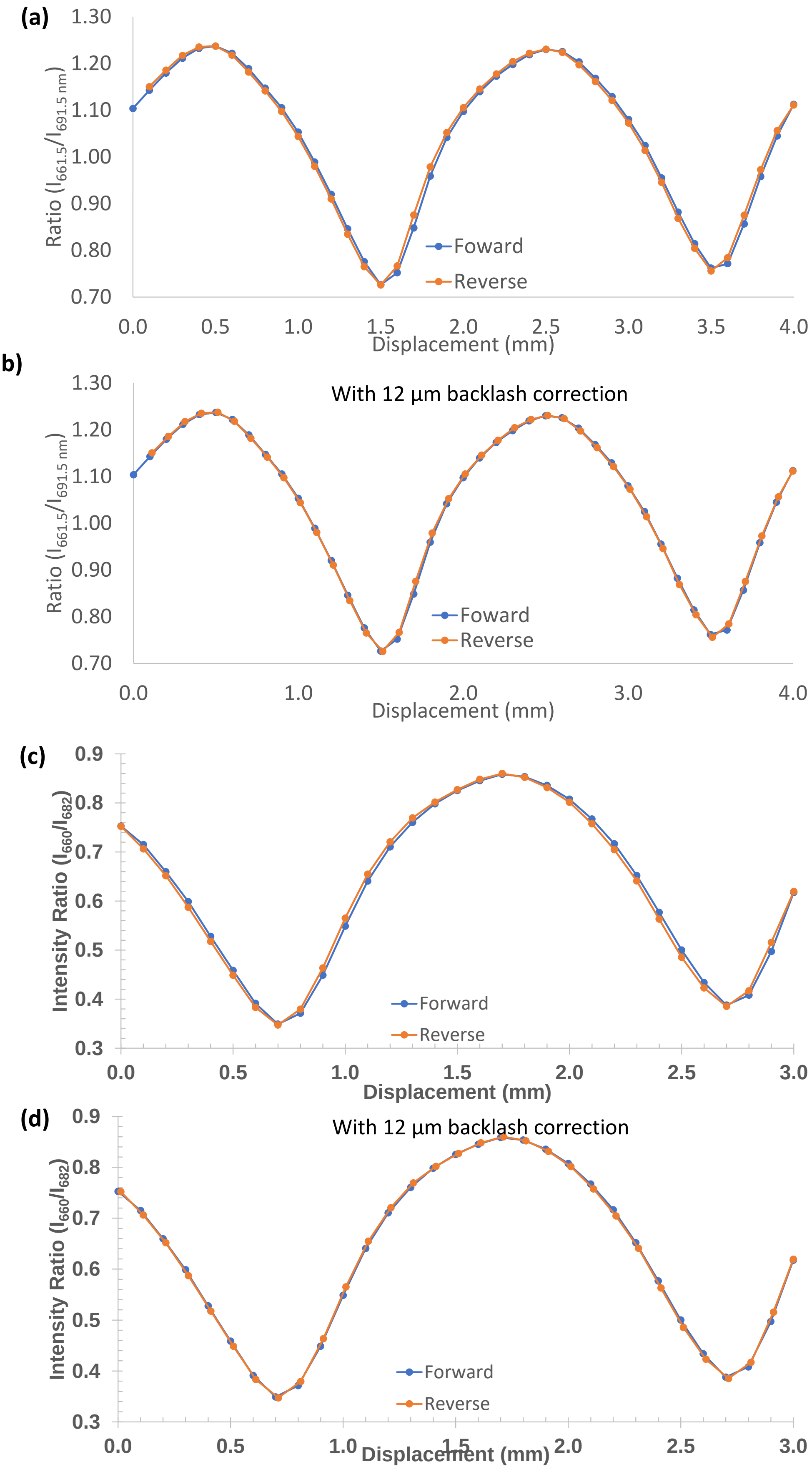

Figure S1. Spectral intensity ratio acquired using a 150 line/mm diffraction grating vs. displacement **(a)** without backlash correction and **(b)** with a 12 µm backlash correction. Spectral intensity ratio acquired using a 1200 lines/mm diffraction grating vs. displacement **(c)** without backlash correction and **(d)** with a 12 µm backlash correction.

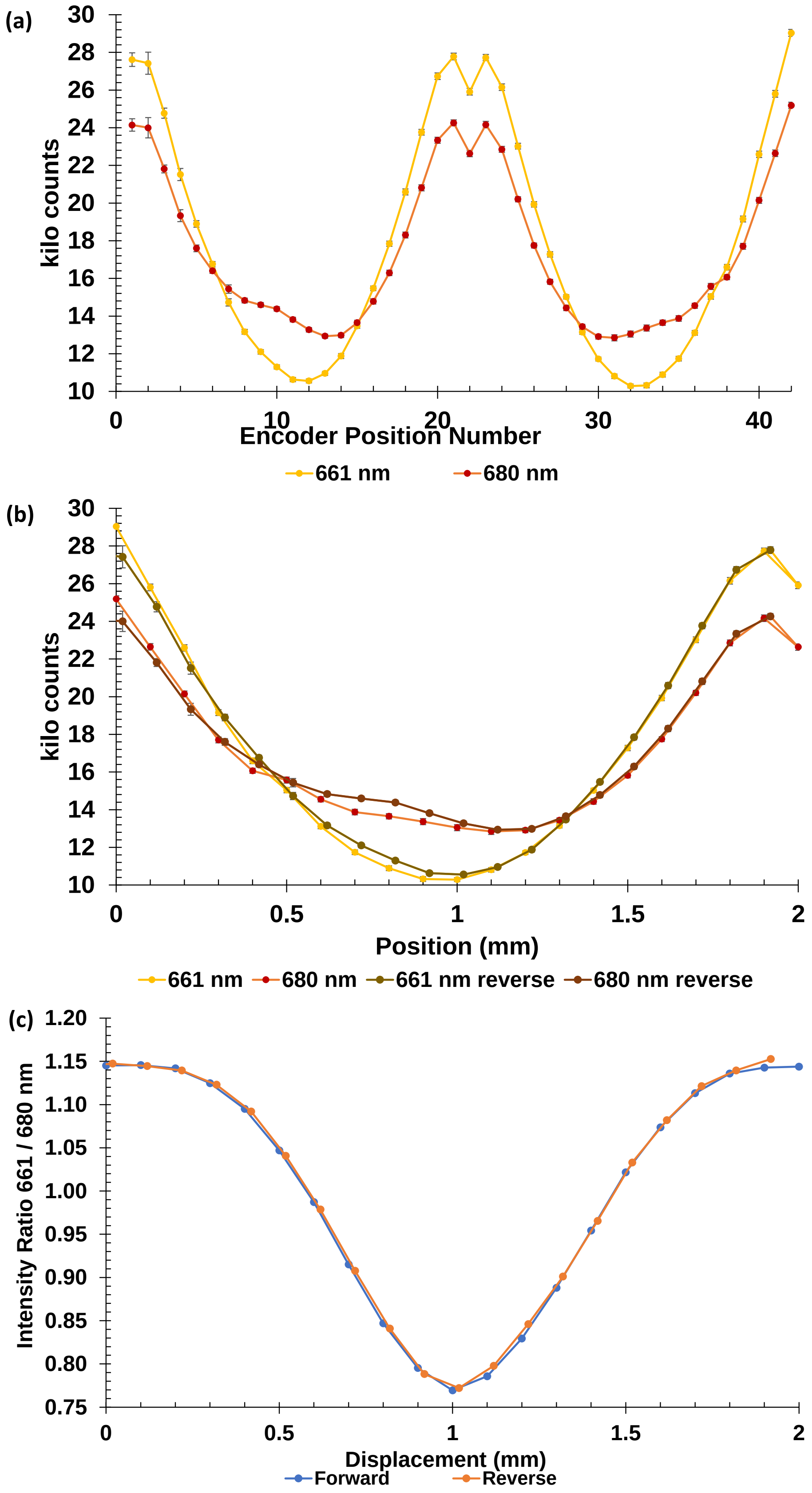

Figure S2. Displacement measurements through 6 mm chicken breast tissue. **(a)** Raw intensities at 661 nm and 680 nm, same data as Figure 5c. **(b)** Replotted as intensity vs. position. **(c)** Calculated Intensity ratio (also shown in Figure 6c).

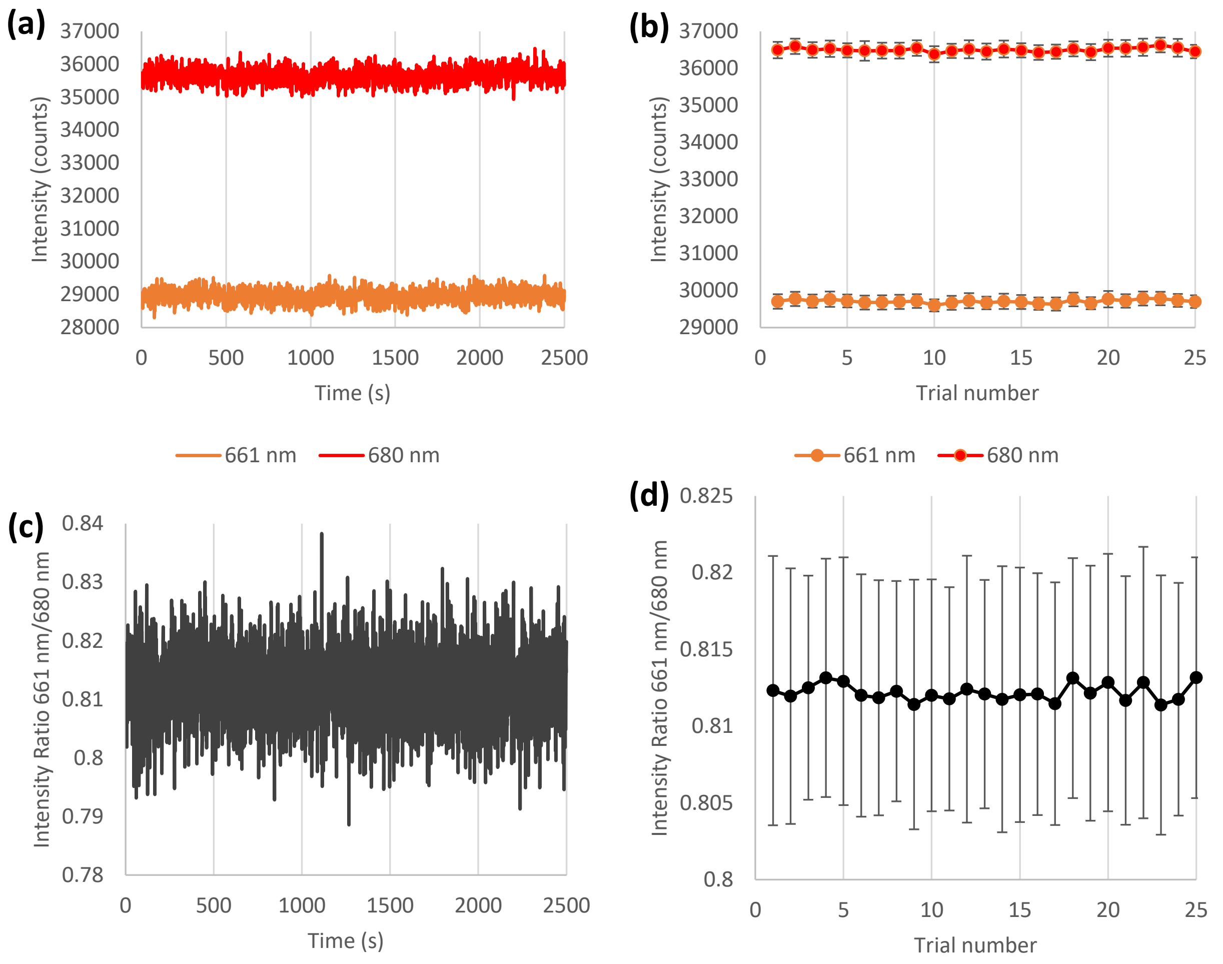

Figure S3. Spectral ruler measurements through 6 mm of chicken tissue, used to generate Figure 5d. **(a)** Intensity at 661 nm and 680 nm (1 s exposure for each measurement). **(b)** Intensity at 661 nm and 680 nm for averaging 100 measurements. **(c)** Ratio of 661 nm/680 nm intensities (1 s exposure for each measurement). **(d)** Ratio of 661 nm/680 nm intensities for averaging 100 measurements.
